# A Comparison of Bone Marrow Morphology and Peripheral Blood Findings in Low and High Level *JAK2* V617F Allele Burden

**DOI:** 10.3390/diagnostics13122086

**Published:** 2023-06-16

**Authors:** Emina Babarović, Blažen Marijić, Luka Vranić, Josipa Ban, Toni Valković, Ita Hadžisejdić

**Affiliations:** 1 Faculty of Medicine, University of Rijeka, 51000 Rijeka, Croatia; esinozic@gmail.com (E.B.); bmarijic@gmail.com (B.M.); luka.vranic@uniri.hr (L.V.); jban9924@gmail.com (J.B.); toni.valkovic@uniri.hr (T.V.); 2 Laboratory for Molecular Pathology, Clinical Department of Pathology and Cytology, Clinical Hospital Center Rijeka, 51000 Rijeka, Croatia; 3 Department of Otorhinolaryngology and Head and Neck Surgery, Clinical Hospital Center Rijeka, 51000 Rijeka, Croatia; 4 Department of Internal Medicine, Clinic for Gastroenterology, Clinical Hospital Center Rijeka, 51000 Rijeka, Croatia; 5 Department of Internal Medicine, Clinic for Hematology, Clinical Hospital Center Rijeka, 51000 Rijeka, Croatia

**Keywords:** *JAK2* V617F, mutation burden, myeloproliferation, megakaryocytes

## Abstract

Cases with low level *JAK2* V617F mutations are increasingly detected; however, the clinical interpretation of the low allele *JAK2* burden may be challenging. The aim of this study is to analyze and compare the bone marrow morphology and peripheral blood findings in the low level *JAK2* V617F allele burden (≤15% of *JAK2*) and high *JAK2* V617F mutation burden patients (>15% *JAK2*). In total, 122 *JAK2* V617F positive cases with concomitant bone marrow biopsies and peripheral blood findings were re-evaluated (62 low and 60 high level *JAK2* V617F positive). Within the low burden group, normal looking megakaryocytes (*p* = 0.0005) were more frequently found, compared with those with no atypia (*p* = 0.0003), their number was more frequently not increased (*p* = 0.009), and they did not form clusters (*p* = 0.001). We found statistically significant difference in the number of platelet (*p* = 0.0003) and hematocrit levels (*p* = 0.032) when comparing the *JAK2* V617F <3% and ≥3% mutation burden. In the high-level burden, the megakaryocytes were more frequently atypical (*p* = 0.054), and more frequently formed clusters (*p* = 0.053) with nuclei with maturation defects (*p* ≤ 0.0001). In conclusion, the *JAK2* V617F mutation burden is reflected by morphological changes in the bone marrow and careful follow up of each and every patient with a low *JAK2* V617F positivity is mandatory.

## 1. Introduction

Philadelphia chromosome negative (Ph-) myeloproliferative neoplasms (MPN) include polycythaemia (PV), essential thrombocytosis (ET), and primary myelofibrosis (PMF). Somatic mutation V617F in Janus kinase 2 (*JAK2*) is the main known diagnostic marker for Ph-MPN resulting in excessive proliferation of one or more myeloid cells [1]. The *JAK2* V617F mutation can be found in more than 95% of patients with PV and 50–60% of patients with ET or PMF [2]. In patients with *JAK2* V617F mutations and allele burden above 50%, there is a higher risk of thrombosis, both in PV and ET [3,4]. On the other hand, studies show that a low *JAK2* V617 allele burden in PMF is linked with statistically significant shorter survival and shorter intervals without progression into acute leukemia [5,6]. The *JAK2* mutation has become a diagnostic standard, and the level of allele burden is part of a usual diagnostic work up, although the World Health Organization (WHO) does not specify the reference value for setting the diagnosis of MPN [7]. Because of the use of allele-specific real time PCR (AS-PCR) with high analytical sensitivity, cases with a low level *JAK2* V617F mutation are increasingly detected; however, the clinical interpretation of the low allele burden of *JAK2* mutation cases may be challenging [8]. Previous studies have shown that a small amount of *JAK2* V617F clone cannot represent sufficient evidence to establish malignant myeloproliferation because clonal hematopoiesis can be present in healthy individuals as well [9]. It is known that 0.03–1% of *JAK2* V617F mutations can also be found in the adult, healthy population [10,11]. The prognostic significance of *JAK2* V617F mutation quantification, as well as the correlation with patient’s clinical characteristics, is not yet fully clarified [12]. Some studies have shown that the burden of *JAK2* V617F alleles can correlate with the phenotypic presentation of MPN [13], severity of the disease phenotype [14], risk of thrombotic events [15], progression to post-PV myelofibrosis, and survival [6,16]. A rare subgroup of patients presenting with splenic vein thrombosis (SVT) frequently present normal or near normal blood counts with small amounts of *JAK2* V617F clone detectable [17]. However, the risk of thrombosis has been shown to increase with the percentage of mutant allele burden and the type of MPN [18]. The *JAK2* V617F mutation is detectable in individuals with clonal haematopoiesis of indeterminate potential (CHIP) and despite normal blood counts, these individuals have a significantly increased risk of cardiovascular disease [19]. Studies imply that when a low positivity of *JAK2* V617F is encountered there is no difference in mutant allele burden and patient characteristics between known MPN cases and reactive cases [20]. In addition, it has also been found that patients with a low-level mutation (*JAK2* V617F < 12%) also had an increased frequency of co-existing mutations in the exon 12 *JAK2* and exon 10 *MPL* genes [21]. Therefore, Wu et al. concluded that in the case of <5% *JAK2* V617F mutations, a low positive result should be reported along with a recommendation for the correlation with the clinical history and bone marrow biopsy findings [20]. This finding is in favor of studies that have shown that bone marrow biopsy, when performed, was diagnostic of full-blown disease, regardless of *JAK2* V617F allele burden [7]. Therefore, the aim of this study is to characterize and analyze the bone marrow morphology and peripheral blood findings in the low level *JAK2* V617F allele burden patients and compare it to the morphology and blood findings of the high *JAK2* V617F mutation burden patients.

## 2. Materials and Methods

This retrospective study included a total of 122 cases referred to the Department of Haematology and to the Clinical Department of Pathology and Cytology, Clinical Hospital Center Rijeka (Rijeka, Croatia), during January 2015 to January 2019. Only cases with available bone marrow biopsy and concomitant results of *JAK2* AS-PCR assay were included. The patients were divided into the low level *JAK2* V617F allele burden group (≤15% of *JAK2*) and high *JAK2* V617F mutation burden group (>15% *JAK2*) with follow up bone marrow biopsy and peripheral blood taken at the time of diagnosis or at the time of JAK2 V617F positivity for comparison. We used 15% as the threshold because the literature states that full blown MPN disease in bone marrow histology is usually 10% and more, so to have substantial number of samples for comparison in our study group, we found 15% JAK2 positivity to be the optimal cut off level of allele burden. The morphological findings of the bone marrow biopsy were re-evaluated at the Clinical Department of Pathology and Cytology, Clinical Hospital Center Rijeka, by two pathologists. A review of the patients’ electronic medical records was performed to collect the complete blood count (CBC) data and clinical characteristics at the time of diagnosis. The patient diagnoses were made locally based on a multidisciplinary integrated review of their clinical, laboratory, and histopathological findings according to the current World Health Organisation (WHO) Classification criteria (2008 or 2016). All of the patients provided written informed consent at the time of clinical work-up and all procedures followed were in accordance with the ethical standards and the Helsinki Declaration. In addition, the study was approved by the Ethics Committees of the Clinical Hospital Center Rijeka and the Faculty of Medicine, University of Rijeka. The general characteristics of the patients are shown in Table 1.

### 2.1. Bone Marrow Biopsy Evaluation

Bone marrow specimens were routinely formalin fixed; decalcified; embedded in paraffin; and stained with H&E, periodic acid-Schiff, Giemsa, Prussian blue, and Gomori to evaluate the morphology and reticulin fiber content. The bone marrow slides were independently evaluated by two pathologists and the following morphologic features were assessed: cellularity, adipocyte percentage and spatial distribution (near trabeculae, diffuse intertarbecular or combination of both), erythroid precursor cells morphology (normoblastic or megaloblastic), myeloid-erythroid ratio (M:E) (reduced, normal, or elevated), reticulin fiber content, and megakaryocyte morphologic features. Cellularity was approximately assessed paralleling an age-associated reduction in the percentage of hematopoietic cells relative to marrow fat [22]. Myelofibrosis was graded on the scale of 0 to 3 according to the European consensus on grading bone marrow fibrosis and the assessment of cellularity [22,23,24]. Megakaryocyte morphologic features (spatial distribution, clustering, morphology, and lobulation of nuclei) were scored for each case using an arbitrary threshold of at least 10% within the cells of a lineage, as described earlier [25]. The bone marrow morphological characteristics and CBC findings were compared between low (<15% of *JAK2* V617F mutation) and high (≥15% of *JAK2* V617F mutation) level allele burden. Furthermore, the low level *JAK2* V617F allele burden group was further subclassified into <3% and ≥3% *JAK2* V617F allele burden groups, and subsequently the morphological and CBC finding were compared.

### 2.2. DNA Isolation and JAK2 V617F Mutation Analysis

The DNA was isolated from the whole blood samples using a Macherey Nagel blood kit (Macherey-Nagel, Duren, Germany) according to the manufacturer protocol. The yield and the quality of isolated DNA was determined using Qubit 3.0 (ThermoFisher, Waltham, MA, USA). The *JAK2* V617F mutation level was evaluated using the ipsogen^®^JAK2 MutaQuant^®^Kit (Qiagen GmbH, Hilden, Germany), using quantitative, real-time AS-PCR with the cobas z480 system (Roche Diagnostics, Rotkreuz, Switzerland). The percentage of mutant *JAK2* V617F allele was expressed as the ratio of *JAK2* V617F copies to the total copy number (CN) of *JAK2*[CN of *JAK2* V617F/(CN of *JAK2* V617F +CN of *JAK2* wild type)] × 100. The cut-off values and interpretation of results were set by the kit’s manufacturer as follows: *JAK2* V617F ≤ 0.014% mutation means not detected, *JAK2* V617F > 0.014% but <0.091% means an inconclusive result (grey zone), and *JAK2* V617F ≥ 0.091% means a positive result and mutation has been detected.

### 2.3. Statistical Analysis

Statistical analysis was performed using MedCalc for Windows, version 20.116 (MedCalc Statistical Software bvba, Ostend, Belgium). Classical methods of descriptive statistics were used, continuous variables were presented as the median and range, and categorical variables were presented as the number of cases and percentage. Differences of continuous variables between analyzed group of patients were done using the Mann–Whitney *U* test. Differences between categorical variables were done using Fisher’s exact test and the χ^2^ test. *p* value < 0.05 was considered statistically significant.

## 3. Results

### 3.1. Diagnoses within Low and High JAK2 V617F Allele Burden Groups

Within the high allele burden group, there was 60 patients in total and the most frequent diagnosis was PV (51.7%), followed by ET (20%) and PMF (18.3%). The bone marrow histology was in the high allele burden group in all cases indicative of MPN. In the low allele burden group, there were 62 patients and the most frequent diagnosis was ET (51.6%) followed by PV (24.2%), unclassified MPN (11.3%), and PMF (which was the lowest in occurrence). Within this group, there was one patient that could be classified as CHIP and three patients had <3% *JAK2* V617F allele burden but did not have MPN, while one patient was diagnosed with chronic myelogenous leukemia (CML). The CHIP patient had 2.04% JAK2 V617F positivity and did not have the abnormal findings in the bone marrow or overt abnormality of CBC. This patient was 75 years old at the time of diagnosis and was lost from the follow up. In addition, the median of JAK2 V617F allele burden in the low positive group (*JAK2* V617F ≤ 15%) was 7.18% (range 0.019–15%) and in the high positive group (*JAK2* V617F > 15%) it was 37.75 (range 15.55–94%) (Table 2).

### 3.2. Comparison of the Bone Marrow Morphology and Peripheral Blood Findings in the Low Level and High Level JAK2 V617F Allele Burden Groups

When comparing the age at diagnosis between the low and high *JAK2* positive groups, there was statistically significantly more patients over 60 years of age in the high *JAK2* group (*p* = 0.007). In addition, when comparing the grade of myelofibrosis between these two groups, there was a slight increase in the frequency of patients with some form of fibrosis in the high positive *JAK2* group and the difference was statistically significant (*p* = 0.023). Normocellular bone marrow was statistically significantly more frequently found in the low positive *JAK2* group than in the high positive *JAK2* group, where we found hypercellular bone marrow more frequently (*p* = 0.005). As a reflection of differences found in the cellularity between these two groups, the proportion of adipocyte was frequently lower in the high *JAK2* positive groups (*p* = 0.009). An increased ratio of myeloid cells was statistically significantly more frequently found in the high *JAK2* positive group (*p* = 0.0012).

The difference in the megakaryocyte clustering and megakaryocyte atypia between the two groups was at the level of statistical trend (*p* = 0.053 and *p* = 0.054, respectively), but we found megakaryocytes forming clusters much less and megakaryocytes with no atypia even less frequently in the low *JAK2* positive group. There was no statistical difference between the low and high *JAK2* groups when we compared the number of megakaryocytes and the erythroid to myeloid cell number ratio. What we found was that there were statistically significantly more frequently staghorn megakaryocytes in the low *JAK2* group, while in the high *JAK2* group, there were more frequently megakaryocytes with a nuclear maturation defect (*p* < 0.0001). When comparing the peripheral blood findings, the statistically significant difference was found in the erythrocyte count and hematocrit level (*p* = 0.0057 and *p* = 0.048, respectively), while the hemoglobin, leucocyte, and platelet count did not show a statistically significant difference between these two groups (Table 3). In addition, in the <3% *JAK2* positive group, we found 11 patients that had less than 1% of mutation burden and 6 patients had normal bone marrow histology. The main characteristic of patients with <3% *JAK2* allele burden are shown in Table 4.

### 3.3. Comparison of the Bone Marrow Morphology and Peripheral Blood Findings within the Low Level JAK2 V617F Allele Burden Group

When comparing the age, there was no statistically significant difference within the low level *JAK2* V617F allele burden group (<3% and ≥3%, but ≤15% in the allele group). In this group, the statistically significant difference was seen when looking the number of megakaryocytes (*p* = 0.009), and in the patient group with <3%*JAK2* V617F, more frequently their count was normal compared with the patients who had >3% mutation burden. In addition, megakaryocyte clustering was much more frequently seen in the ≥3% but <15% allele group (*p* = 0.001).

**Figure 1:**
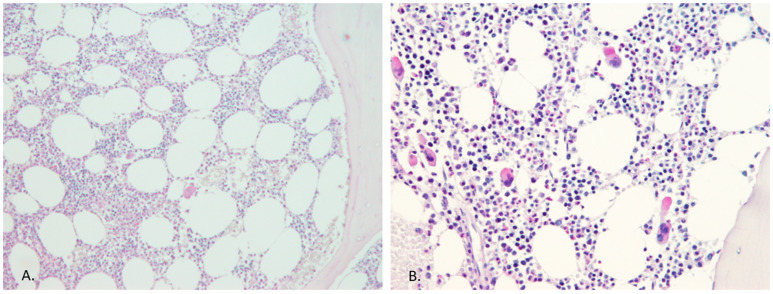
Megakaryocyte count and morphology within the low level *JAK2* burden group. Bone marrow histology, in the case of the high *JAK2* allele burden, always confirmed the MPN diagnosis, but in the case of a low allele burden (<3% of *JAK2* allele burden), it was not helpful in all instances. (**A**) This patient had 0.27% *JAK2* V617F within the peripheral blood and the bone marrow morphology was normal (PAS staining, ×10 magnification). (**B**) This image shows bone marrow with a slight increase in megakaryocyte count but with no atypia, and the patient was 0.54% positive for the *JAK2* V617F mutation (PAS staining, ×20 magnification).

When comparing the megakaryocyte nuclear morphology there was much more normal looking megakaryocyte nuclei in the <3% allele group, while staghorn looking nuclei were more frequently seen in the ≥3% but <15% allele group (*p* = 0.0005) (Figure 1). In addition, the megakaryocyte nuclear atypia was more frequently found when the *JAK2* V617F mutation burden was higher than 3% (*p* = 0.0003). In the comparison of peripheral blood findings, there was a statistically significant difference between two groups when the platelet count and hematocrit level were compared (*p* = 0.0003 and *p* = 0.032, respectively), while the hemoglobin was at the level of statistical trend (*p* = 0.058). The erythrocyte and leucocyte count did not show statistically significant difference between these two groups (Table 5).

In all of the examined groups, we did not find statistically significant associations between the *JAK2* V617F mutation burden and thrombotic events (data not shown).

## 4. Discussion

After the introduction of sensitive assays, such as AS-PCR, low level *JAK2* V617F mutation burden cases were more frequently detected. Studies indicate that majority of cases with MPN have an overt positivity (>10%) of *JAK2* V617F mutation, while in healthy individuals the mutational load is typically <1% of *JAK2* V617F, with rare exemptional cases [20]. The detection of *JAK2* V617F is not sufficient for a diagnosis of Ph-MPN, so the clinical interpretation of low level *JAK2* V617F allelic burden is challenging. In this study, the majority of patients in the high level *JAK2* V617F (>15% *JAK2*) positive group were diagnosed with PV, while in the low level *JAK2* V617F positive group (≤15% of *JAK2*), the most frequent MPN was ET (Table 2). These findings are in agreement with the study of Nielsen et al., who showed that the *JAK2* V617F mutation burden level was associated with MPN disease development and progression rate, consistent with a biological continuum of increasing the *JAK2* V617F allele burden across the growing severity of myeloproliferative neoplasm from no disease through ET and PV to PMF [26]. In this study, within the low allelic burden group, normal looking megakaryocytes were more frequently found, compared with no atypia, their number was more frequently not increased, and they did not form clusters. These findings indicate that in the portion of low positive *JAK2* V617F cases, the bone marrow morphology will be normal. This might be either be because the patient’s bone marrow biopsy was taken at the early stage of disease or the patient had a mild form of disease with only minor histological features that were insufficient to confirm MPN. Considering that bone marrow biopsy is an aggressive diagnostic procedure, in the low *JAK2* V617F mutation burden patients, the more appropriate approach for the patient diagnostic workup would be to order a complete blood count (CBC), LDH, and abdominal ultrasound, and after 12 months to re-test the *JAK2* V617F mutation status. Therefore, the management of low positive *JAK2* V617F patients needs to be individualized based on their age, other comorbidities, having risk factors for thrombosis, etc. In this study, within the low-level mutation burden group (<3% of *JAK2* V617F), we found one case of CHIP and three patients did not have MPN. Perricone et al. suggested that a cut-off of ≥0.8% *JAK2* V617F allele burden is very indicative for the presence of MPN and that monitoring the *JAK2* mutation burden over time is a convenient way to assess clonal hematopoiesis expansion [7]. However, some studies have shown that *JAK2* V617F allele burden can be present and can increase in individuals with no evidence of MPN [9]. However, Nielsen et al. suggested a 2% cut-off value for disease versus no disease for *JAK2* V617F positive individuals [26]. In addition, some found that the ≥2% cut-off for *JAK2* V617F allele burden was of clinical interest as such patients were more likely to present with splenomegaly and evolve towards MPN within first year of follow up [27]. However, even individuals with a *JAK2* V617F mutation burden below 2% should receive medical attention as in time, many of them will develop a myeloproliferative neoplasm indicating the presence of a latent form of Ph-MPN [26]. Therefore, a careful follow up of every patient with a low positive *JAK2* V617F mutation allele burden is mandatory, especially if bone marrow histology does not confirm MPN diagnosis. There is substantial doubt about the prognosis in *JAK2* V617F positive individuals without evident signs of myeloproliferative disease. CHIP belongs to a spectrum of hematological pre-malignant states and is associated with the development of various hematological malignancies; however, most carriers do not develop malignancy and the progression rate is approximately 0.5–1% per year [28,29]. In general, the population with CHIP have a 40% higher mortality than those without CHIP, and this is reflected by striking excess of cardiovascular events [30]. Some authors have also suggested that testing for latent MPN should be performed in cases of cerebral vein thrombosis [31]. It has been shown that risk of thrombosis increases with the percentage of mutant allele burden and the type of MPN [27]. In this study, there was statistically significant difference in the number of platelet and hematocrit level when comparing two *JAK2* V617F mutation burden groups, <3% and ≥3%. In the high-level allelic burden (when the cut-off for comparison was ≥3% and >15%), the megakaryocytes were more frequently atypical, more frequently forming clusters with the nuclei having a maturation defect. These findings indicate that the *JAK2* mutation burden is reflected by morphologic changes in the bone marrow, mostly found within the megakaryocytes.

In this study, we found one patient within the low level *JAK2* V617F group that could classify as CHIP, indicating that CHIP does exist (and as studies indicate it may be linked to significant morbidity). However, we also found 11 cases of *JAK2* V617 mutation burden below 1% and 3 of them did not have MPN. Taking into account that thrombosis is the main cause of morbidity and mortality in patients with *JAK2* V617 MPN, the clinically relevant cut-off level of *JAK2* mutation burden to start prophylactic anticoagulation remains to be determined, i.e., >0% vs. ≥2%. It has been shown that JAK2 inhibitory therapy in MPN has a limited disease modifying potential. Current JAK2 inhibitors are used in the case of intermediate or high-risk PMF in the patients with PV resistance or intolerance to hydroxyurea, while patients with ET did not have an additional benefit [32]. The reduction in the mutant allele burden with JAK2 inhibitory therapy is modest and it has been shown that there might be a clonal evolution progression during treatment, so low JAK2 positive cases certainly will not benefit from it.

In conclusion, the *JAK2* V617F mutation burden is reflected by morphological changes in the bone marrow and careful follow up of each and every patient with a low *JAK2* V617F positivity is mandatory.

## Figures and Tables

**Table 1:** General patient characteristics at the time of diagnosis.

Demographics	Low *JAK2* V617F		High *JAK2* V617F
	<3% (*n* = 15)	≥3% but ≤15% (*n* = 47)	>15% (*n* = 60)
Mean age, years (range)	57 (28–91)	61 (23–86)	69 (39–91)
Subjects aged ≤60 years, *n* (%)	9 (60)	19 (40.4)	13 (21.7)
Subjects aged >60 years, *n* (%)	6 (40)	28 (59.6)	47 (78.3)
Male, *n* (%)	8 (53)	16 (34)	23 (38)
Female, *n* (%)	7 (47)	31 (66)	37 (62)
Splenomegaly, *n* (%)	1 (6.7)	9 (19.1)	18 (30)
Hepatomegaly, *n* (%)	1 (6.7)	2 (4.3)	2 (3.3)
Hepatosplenomegaly, *n* (%)	1 (6.7)	2 (4.3)	6 (10)
Thrombotic events *, *n* (%)	2 (40)	10 (21.3)	12 (20)
JAK2 V617F allele burden, median (range) %	0.41 (0.019–2.04)	9 (3.10–15)	37.75 (15.55–94)

* Thrombotic events at the time of diagnosis and follow up.

**Table 2:** Final diagnoses within low and high level JAK2 V617F positive groups.

Characteristic	JAK2 V617F Allel Burden, Median (Range) %	PV	ET	PMF	Unclassified MPN	CHIP	Other	Total
<3%		3	4	0	3	1	4	15
≥3%		12	28	3	4	0	0	47
Low ≤ 15% *n* (%)	7.18 (0.019–15)	15 (24.2)	32 (51.6)	3 (4.8)	7 (11.3)	1 (1.6)	4 (6.5) *	62
High > 15% *n* (%)	37.75 (15.55–94)	31 (51.7)	12 (20)	11 (18.3)	5 (8.3)	0	1 (1.7) ^&^	60

CHIP = clonal haematopoiesis of an indeterminate potential. * one CML and two were not MPN, ^&^ MPN/MDS.

**Table 3:** Evaluation of bone marrow morphology and peripheral blood characteristics of low (≤15%) versus high (>15%) *JAK2* V617F positive groups.

Low ≤15% and High >15% *JAK2* V617F Positive *n* = 122			
Characteristic	≤15% *n* = 62	>15% *n* = 60	*p* Value
Age (years) *n* (%) ≤60 >60	28 (45.2) 34 (54.8)	13 (21.7) 47 (78.3)	**0.007** *
Myelofibrosis 0 1 2 3	46 (74.2) 12 (19.4) 4 (6.5) 0 (0)	31 (51.7) 15 (25) 11 (18.3) 3 (2.5)	**0.023** *
Cellularity Decreased Normal Increased	0 (0) 29 (46.8) 33 (53.2)	1 (1.7) 12 (20) 47 (78.3)	**0.005** *
Adipocytes Normal Decreased	30 (48.4) 32 (51.6)	43 (72.9) 16 (27.1)	**0.009** *
Erythroid cells ratio Decreased Normal Increased	9 (14.5) 15 (24.2) 38 (61.3)	10 (17.2) 6(10.3) 42 (72.4)	0.137 *
Myeloid cells ratio Decreased Normal Increased	10 (16.1) 30 (48.4) 22 (35.5)	7 (11.9) 12 (20.3) 40 (67.8)	**0.0012** *
E:M ratio E > M E = M E < M	22 (35.5) 21 (33.9) 19 (30.6)	19 (32.8) 18 (31) 21 (36.2)	0.812 *
Number of megakaryocytes Decreased Normal Increased	1 (1.6) 9 (14.5) 52 (83.9)	0 (0) 4 (6.7) 56 (93.3)	0.219 *
Megakaryocytes clustering Yes No	43 (69.4) 19 (30.6)	51 (85) 9 (15)	**0.053** *
Megakryocyte nucleus morphology Normal Hypolobulated Staghorn Maturation defect	9 (14.5) 8 (12.9) 32 (51.6) 13 (21)	0 (0) 1 (1.7) 23 (39.0) 35 (59.3)	**<0.0001** *
Megakryocyte atypia Yes No	53 (85.5) 9 (14.5)	58 (96.7) 2 (3.3)	**0.054** *
RBC, median (range) ×10^12^/L	5.10 (1.19–6.67)	5.51 (2.40–8.57)	**0.0057** ^&^
Hemoglobin, median (range) g/L	147 (70–191)	153 (57–229)	0.1607 ^&^
Hematocrit, median (range) %	0.455 (0.206–0.586)	0.482 (0.23–0.74)	**0.048** ^&^
WBC, median (range) ×10^9^/L	9.3 (4.8–20.6)	10.15 (4.1–34.8)	0.070 ^&^
PLT, median (range) ×10^9^/L	557 (34–1131)	513 (117–1604)	0.993 ^&^

E:M, erythroid-myeloid ratio; RBC, red blood cells; WBC, white blood cells; PLT, platelet count; * Fischer’s exact test; ^&^ Mann-Whitney test.

**Table 4:** Main characteristic of patients within the <3% *JAK2* V617F allele burden group.

Subject	%*JAK2* V617F	Bone Marrow Histology	RBC	WBC	PLT	Final Diagnosis
S1	2.04	Normal	N	C	N	CHIP/old
S2	0.12	Suggestive of MPN	Slightly increased	N	N	Probable MPN (PV?)
S3	0.41	Suggestive of MPN	Slightly increased	N	N	Probable MPN (PV?)
S4	0.23	MPN	N	N	N	Unclassified MPN
S5	0.54	Normal	N	N	Slightly increased	Probable ET
S6	0.41	MPN	N	N	Increased	ET
S7	1.07	MPN	N	N	Increased	ET
S8	1.49	MPN	Slightly increased	N	N	Probable MPN (PV?)
S9	0.27	Normal	N	N	Slightly increased	Probable ET
S10	0.56	Normal	N	N	N	Not MPN
S11	0.019	Normal	No data	No data	No data	Not MPN
S12	0.25	CML	N	Increased	Decreased	CML
S13	1.20	Suggestive of MPN	Slightly increased	N	N	Probable MPN
S14	0.11	Suggestive of MPN	Slightly increased	N	N	Probable MPN
S15	0.12	Normal	Slightly increased	N	N	Not MPN

RBC, red blood cells; WBC, white blood cells; PLT, platelet count; MPN, myeloproliferative neoplasm; N, normal; CML, chronic myelogenous leukemia; PV, polycythaemia vera; ET, essential thrombocytosis.

**Table 5:** Bone marrow morphology and peripheral blood characteristics for the low *JAK2* V617F (≤15%) positive group.

Low *JAK2* V617F Positive ≤15% *n* = 62			
Characteristic	<3% *n* = 15	≥3% and ≤15% *n* = 47	*p* Value
Age (years) *n* (%) ≤60 >60	9 (60) 6 (40)	19 (40.4) 28 (59.6)	0.238 *
Myelofibrosis 0 1 2	11 (73.3) 3 (20) 1 (6.7)	35 (74.5) 9 (19.1) 3 (6.4)	0.996 *
Cellularity Normal Increased	10 (66.7) 5 (33.3)	19 (40.4) 28 (59.6)	0.136 *
Adipocytes Normal Decreased	10 (66.7) 5 (33.3)	22 (46.8) 25 (53.2)	0.239 *
Erythroid cells ratio Decreased Normal Increased	2 (13.3) 3 (20) 10 (66.7)	7 (14.9) 12(25.5) 28 (59.6)	0.879 *
Myeloid cells ratio Decreased Normal Increased	3 (20) 8 (53.3) 4 (26.7)	7 (14.9) 22 (46.8) 18 (38.3)	0.699 *
E:M ratio E > M E = M E < M	7 (46.7) 4 (26.7) 4 (26.7)	15 (31.9) 17 (36.2) 15 (31.9)	0.576 *
Number of megakaryocytes Decreased Normal Increased	1 (6.7) 5 (33.3) 9 (60)	0 (0) 4 (8.5) 43 (91.5)	**0.009** *
Megakaryocytes clustering Yes No	5 (33.3) 10 (66.7)	38 (80.9) 9 (19.1)	**0.001** *
Megakryocyte nucleus morphology Normal Hypolobulated Staghorn Maturation defect	7 (46.7) 2 (13.3) 3 (20) 3 (20)	2 (4.3) 6 (12.8) 29 (61.7) 10 (21.3)	**0.0005** *
Megakryocyte atypia Yes No	8 (53.3) 7 (46.7)	45 (95.7) 2 (4.3)	**0.0003** *
RBC, median (range) ×10^12^/L	5.19 (4.12–6.67)	5.02 (1.91–6.06)	0.345 ^&^
Hemoglobin, median (range) g/L	152.5 (113–191)	145 (70–175)	0.058 ^&^
Hematocrit, median (range) %	0.48 (0.35–0.58)	0.45 (0.20–0.53)	**0.032** ^&^
WBC, median (range) ×10^9^/L	9.65 (6.4–20.6)	9.1 (4.8–15.3)	0.643 ^&^
PLT, median (range) ×10^9^/L	265 (34–1008)	602 (157–1131)	**0.0003** ^&^

E:M, erythroid–myeloid ratio; RBC, red blood cells; WBC, white blood cells; PLT, platelet count; * Fischer’s exact test; ^&^ Mann–Whitney test.

## Data Availability

The datasets generated and analyzed during the current study are available from the corresponding author upon reasonable request.
